# Real versus sham proximal biofield therapy in the treatment of warts of the hands and feet in adults: study protocol for a randomized controlled trial (MAGNETIK study)

**DOI:** 10.1186/s13063-017-1994-4

**Published:** 2017-06-07

**Authors:** Cathy Gaillard, Laure Allain, Hélène Legros, Sylvie Brucato, Yohann Desgue, Christophe Rouillon, Laure Peyro-Saint-Paul, Anne Dompmartin

**Affiliations:** 1 Délégation à la Recherche Clinique et à l’Innovation, Centre Hospitalier Régional Universitaire (CHU) de Caen, Avenue de la Côte de Nacre, CS 30001, F-14000 Caen, France; 2 Centre de Recherche Clinique, Centre Hospitalier Régional Universitaire (CHU) de Caen, Avenue de la Côte de Nacre, CS 30001, F-14000 Caen, France; 3 Biofield Therapy Center, 17 rue des ormes, F-50570 Marigny, France; 4 Service de Dermatologie, Centre Hospitalier Régional Universitaire (CHU) de Caen, Avenue de la Côte de Nacre, CS 30001, F-14000 Caen, France

**Keywords:** Biofield therapy, Alternative medicine, Warts, Papillomavirus infections, Randomized controlled trial, Single-blind and assessor-blind method

## Abstract

**Background:**

Despite the lack of scientific studies on biofield therapies, they are widely acclaimed by patients. The mechanisms of action are not explained by current allopathic medical approaches. Warts are common and contagious viral lesions that may be refractory to standard dermatologic treatments such as cryotherapy, laser therapy, and keratolytic ointments. Biofield therapies are efficient in various pathologies. Their ability to treat warts has never been demonstrated in a scientific study with a robust methodology. Patients with refractory warts often place their trust in these alternative therapies because of the poor results obtained from traditional medicine. We propose a prospective, randomized, single-blind, assessor-blind trial to evaluate the efficacy of treatment of warts by biofield therapy.

**Methods/design:**

Subjects with warts on their feet or hands will be randomized into two groups: real biofield therapy versus sham therapy. The diagnosis will be made at the time of inclusion, and follow-up will take place in week 3. Comparison of pictures of the warts at baseline and after 3 weeks will be used as the primary outcome measure. The hypothesis is that the extent of the disappearance of the original wart in the group treated by real biofield therapy will be 70% and that it will be 30% in the group treated by sham therapy. Using 90% power and an alpha risk of 5%, 31 subjects are required in each group for a two-tailed proportion comparison test.

**Discussion:**

To our knowledge, this is the first study to evaluate the efficacy of biofield therapy on warts. Therefore, the aim of this study is to extend knowledge of biofield therapy to another area of medicine such as dermatology and to propose complementary or alternative practices to improve patient well-being. The main strength of the study is that it is a randomized, single-blind, assessor-blind, placebo-controlled study.

**Trial registration:**

ClinicalTrials.gov identifier: NCT02773719. Registered on 22 April 2016.

**Electronic supplementary material:**

The online version of this article (doi:10.1186/s13063-017-1994-4) contains supplementary material, which is available to authorized users.

## Background

The concept of biofield therapies originates from many different cultures over thousands of years [1]. Currently, biofield therapies are used increasingly in modern-day healthcare and have only recently been studied by conventional scientific methods to evaluate their actual effects. Biofield therapies are defined as noninvasive, practitioner-mediated practices that stimulate the healing response in patients. Biofield therapies show strong evidence of reducing pain intensity in pain populations and moderate evidence of reducing pain intensity in hospitalized and cancer patient populations [2]. Authors of a recent review [3] reported 18 clinical trials on biofield therapies with a high level of evidence, among which 12 had at least one primary outcome with statistically significant beneficial treatment. More moderate evidence in a nonrandomized study showed a decrease in depressed mood [4] or reduced pain after biofield therapies in populations with cancer [5]. These healing practices involve electromagnetic fields that are delivered either proximally (with the practitioner and the receiver in the same room) or distally. Biofield therapy produces a wide variety of clinically significant effects, including growth enhancement, wound repair, regeneration, and reduction of pain [6–10], but, to our knowledge, nothing has been studied to date in the treatment of warts. Palmar or plantar warts are one of the most common infectious skin diseases caused by the human papillomavirus. Even if warts are benign skin growths that disappear over time without treatment, they remain unsightly and contagious and can cause significant discomfort. The main topical treatments for cutaneous warts are summarized in a Cochrane review [11]. Salicylic acid and cryotherapy stand out as the most commonly used treatments, but they remain only moderately effective. Resistance of warts to conventional therapies leads some patients to use alternative medicine such as biofield therapies, and most of them report positive results. At this stage, however, there is no clear or convincing scientific evidence to prove a positive effect. Despite controversies and current gaps in research studies, biofield therapies are widely used by a significant number of patients. Some believe that this response derives partly from the placebo effect.

The purpose of this study is to objectively determine the impact of biofield therapies on wart treatment after a single session according to the main recommendations of clinical studies on biofield therapies [12]. To this end, we designed a study protocol with the appropriate methodology in which patients are treated either by a certified biofield therapist or a sham therapist who mimics all procedures used by the biofield therapist (i.e., placebo group).

## Methods/design

### Study design

This protocol was developed in accordance with the Standard Protocol Items: Recommendations for Interventional Trials (SPIRIT) Statement. For the SPIRIT checklist see Additional file 1. The MAGNETIK study is a prospective, randomized, placebo-controlled, single-blind, assessor-blind trial. The purpose of this study is to evaluate the benefit of biofield therapies on common feet or hand warts when practiced by a real or placebo practitioner.

### Study population

**Fig. 1:**
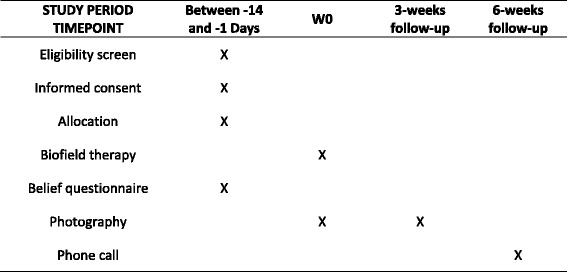
Schedule of enrollment, interventions, and assessments

A total of 62 subjects will be included in this trial. Thirty-one patients will receive biofield therapy delivered by a certified practitioner, and 31 patients will be assigned to the placebo arm with a sham therapist. The participant timeline is shown in Fig. 1.

### Eligibility

Patients with common warts on the hands or feet for at least 3 months are eligible for the study. Patients meeting one or more of the following criteria will not be included:

- Lack of informed consent prior to randomization
- Less than 18 years old or under legal protection
- Treated with oral corticotherapy for more than 6 months
- Immunosuppressed or with a history of transplant surgery
- Undergoing chemotherapy or suspected to have cancerous warts
- Patients with infected warts; injured warts; already treated for 90 days with chemical processes, medical devices, or surgery; or already having received biofield therapy

Subjects will be excluded from the study if their warts are treated with other treatment during the protocol. The appropriate examinations will be performed to ensure that subjects meet all the criteria. The subjects are free to leave the study at any time. Occurrence of adverse events can also lead to discontinuation.

### Recruitment

Subjects will be recruited by an advertising and information campaign at dermatologists’ practices and general practitioners’ practices in or near Caen, France.

### Random allocation

Randomization will be performed using an interactive web response system with Ennov Clinical® Software (Ennov, Paris, France). Trial participants and dermatologists assessing outcomes are blinded to allocation; however, the study nurses and sponsor are not.

### Study protocol

All study procedures take place at the clinical research unit in Caen University Hospital, Caen France. First (i.e., at enrollment), after written informed consent is obtained, the dermatologist establishes a diagnosis of warts on the feet and/or hands and then performs mapping to record warts one by one. Each wart is individually numbered to correlate measurements (baseline vs. W3) or original wart designation (*see below*) to the initial mapping. Before the intervention (W0), each wart is photographed (baseline picture) by the study nurse in accordance with location and number label drawn on the map. The subject is interviewed about beliefs (i.e., by questionnaire) about biofield therapies and is treated according to randomization by either the real or the sham practitioner. The intervention consists of a single session of biofield therapy performed by a real or sham therapist and usually takes about 40 minutes.

The French organization for alternative medicines (the Groupement national pour l’organisation des médecines alternatives [GNOMA]) was asked to validate the skills of the study practitioners. For this purpose, two independent experts from the GNOMA attended a training session. They concluded on the biofield therapeutic abilities of the real practitioner and confirmed the sham therapist, also known as the placebo, to have no biofield therapeutic abilities. The placebo therapist learned the movements during training sessions in order to reproduce the gestures used by the real practitioner.

When the therapist meets the subject (W0), he or she assesses the subject’s level of energy and asks the subject what the subject perceives while (1) facing the subject, with hands placed at either side of the subject’s face (the therapist expects the subject to feel heat or dizziness); (2) with hands placed above the shoulders in an attempt to rock the subject forward and backward; and (3) with hands placed on the middle of the subject’s back (heat should be felt), then on the lower back (cold should be felt). If the subject’s perception is too poor, meaning the subject’s energy level is too low, the therapist then magnetizes the plexus area in a horizontal line before treating the warts. When the subject’s energy level has been assessed, the wart is treated.

The therapist first magnetizes using the index finger placed on top of each wart. Where there are multiple warts, the therapist identifies the original wart and, with the other hand, follows the path of the wart, hooks a finger above it while saying that the wart has been caught, and asks if the subject can feel it. The therapist then pulls on the wart with the finger, as if pulling a thread, until the subject says he or she no longer feels anything. At the end of the session, the therapist checks that the subject no longer feels anything and provides the subject with some recommendations concerning the wart, such as avoiding contact with water or avoiding physical exercise within the next 24 h. The therapist explains that, after few days, a black thread will appear on the wart. If this occurs, the patient is asked to cover it with a plaster.

The subject then returns home and comes back to the clinical research unit for a follow-up visit 3 weeks later. The 3-week visit is scheduled to assess whether the warts have disappeared. Another picture is taken by a study nurse in the same conditions as those at baseline. The subject is interviewed about any treatments taken during this period. Patients are excluded if they use any wart treatment. Six weeks postintervention (W6), the subject will be contacted by a study nurse to assess wart disappearance and to ensure that no medical wart treatments were used. All interventions and assessment time points are presented in Fig. 1.

### Adverse events

Information on all adverse events, such as pain, blistering, and scarring, will be collected and reported to the sponsor. Events will be analyzed by both the investigator and the sponsor and will be qualified as serious or not and related or not.

### Primary outcome measurement

The primary outcome is the disappearance of the original wart between W0 and W3. The dermatologist qualifies each wart—the original wart and the others—without knowing which one is designated by the therapist as being the original one. The study nurse then decodes which is the original wart and collects the data in the electronic case report form. At the end of the study, pictures of warts at W0 and W3 are evaluated by a blinded dermatologist using Mesurim® software to measure the mean diameter of each wart numbered and mapped. Data are recorded in a separate file with a picture code only. The percentage reduction in each wart will be calculated using the mean diameter of each wart at W0 and W3 by the data analyst.

### Secondary outcome measurements

The first secondary objective is the disappearance of all warts other than the original wart 3 weeks after the therapy. Disappearance of warts at 6 weeks will be investigated by making a telephone call to the subject. The subject’s level of belief in biofield therapy at W0 is recorded (believer, skeptical, nonbeliever).

### Data management

Data will be collected and anonymously registered using electronic case report forms in Ennov Clinical® software.

### Data monitoring

The medical procedures used in this trial comply with the most recent recommendations of the Declaration of Helsinki and French Public Health Law 2004-806 of 9 August 2004 on subject protection and safety in accordance with good clinical practice. A person mandated by the sponsor will ensure monitoring of this trial to guarantee that accurate, full, and reliable data are collected. The level of monitoring will be adapted to the low risk of the study.

At the end of the study, the data review committee, comprising the data manager, the biostatistician, and an independent dermatologist, will review all deviations from the protocol. Other members may join the committee to provide some details on the context of each deviation. The committee will qualify deviations as major or minor and shall clarify the relevance of the data with respect to these deviations: conservation of the data (for minor deviation) or exclusion of the data (for major deviation). Major deviations can affect subject safety or rights. Also, by definition, the intention-to-treat analysis requires that all data be kept for the analysis, even major deviations, except in the event of absence or withdrawal of written consent that systematically results in the exclusion of any data on the research.

### Sample size calculation

Sample size was calculated using the observed proportions method (arcsin approximation) with an open source online tool for calculating sample size by power and alpha risk. The hypothesis was that the extent of the disappearance of the original wart in the group treated by the real biofield therapist would be 70% and that it would be 30% in the group treated by the sham therapist. Indeed, it is estimated that one-third of warts resolve spontaneously within 6 months and that spontaneous regression occurs in two-thirds of cases within 2 years. Using 90% power and an alpha risk of 5%, 31 subjects are required in each group for a two-tailed proportion comparison test.

### Statistical methods

Descriptive analyses with results expressed as mean and SD will be used to describe the characteristics of the participants. Categorical variables will be described as frequency and 95% CI. The primary outcome analysis will follow the intention-to-treat principle, in which all randomized patients will be analyzed in the assigned group. Both arms will be compared using Fisher’s exact test or Pearson’s chi-square test for heterogeneity analysis or Student’s *t* test accordingly. No interim analysis is anticipated. All statistical analyses will be conducted using SAS version 9.4 software (SAS Institute, Cary, NC, USA). Statistical significance will be assumed as *p* < 0.05.

### Dissemination protocol

According to the Standard Protocol Items: Recommendations for Interventional Trials (SPIRIT) guidelines, the authors declare that if the blinding is lifted, data will not be presented prior to release of the main results. The blinding will be lifted only at the end of the study. A clinical article will be written on the primary and secondary outcomes of the study. This trial is not industry-sponsored; therefore, there are no publication restrictions imposed by the funding institutional organization.

## Discussion

The goal of our study is to evaluate the efficacy of proximal therapies provided by a biofield therapist to heal cutaneous warts. The study may confirm the promising results of this treatment without furthering understanding of the mechanisms of action.

The first phase is a study protocol with a high level of evidence that could lead to strong conclusions. The main strength of the study design is that it is a single-blind, assessor-blind, placebo-controlled, randomized study.

The second phase, looking at mechanisms, will be explored only if efficacy is proven. The results of our study protocol could provide material to put forward hypotheses, to conduct new studies, and to eventually elucidate testable theories.

The biofield therapy approach to wart treatment will be discussed, as will which professionals are qualified to offer such treatment. Data derived from trials on salicylic acid and cryotherapy show only a moderate therapeutic effect [11]. Other therapies are anecdotal and could not be recommended. It may become a first-line therapy because it is less invasive than conventional treatments. To our knowledge, this is the first study to evaluate the efficacy of biofield therapy on healing cutaneous warts with a robust methodology.

### Trial status

The first participant was recruited in April 2016, and the study is currently enrolling participants. The study was expected to be completed in December 2017.

## Additional files

Additional file 1: SPIRIT checklist. (DOC 122 kb)

Additional file 2: Information document and Consent form (in French, approved by competent authorities). (PDF 347 kb)

## Abbreviations

GNOMA: Groupement national pour l’organisation des médecines alternatives
SPIRIT: Standard Protocol Items: Recommendations for Interventional Trials
