# Supplementary material for: Real versus sham proximal biofield therapy in the treatment of warts of the hands and feet in adults: study protocol for a randomized controlled trial (MAGNETIK study)
Source: Trials. 2017 Jun 7;18:263. doi: 10.1186/s13063-017-1994-4 (PMC5463300; doi:10.1186/s13063-017-1994-4)
Supplement: Supplementary file 2 — Information document and Consent form (in French, approved by competent authorities). (PDF 347 kb) [file 13063_2017_1994_MOESM2_ESM.pdf]

Document d'information du patient

Madame, Monsieur,

Le Docteur ……………………… vous propose de participer à une recherche biomédicale intitulée : ***« Protocole MAGNETIK - Effet de l'intervention d'un magnétiseur sur les verrues des mains et des pieds des adultes : essai prospectif randomisé versus placebo en double aveugle »***

Le promoteur est le CHU de Caen, avenue de la côte de nacre – 14033 CAEN, téléphone : 02.31.06.54.73.

L'investigateur coordinateur de l'étude est le Dr Anne DOMPMARTIN, service de dermatologie au CHU de Caen, avenue de la côte de nacre – 14033 CAEN, téléphone : 02.31.27.25.10.

L'étude clinique est référencée sous le numéro **ID RCB n°2015-A01609-40**.

Le promoteur de l'étude a souscrit une **assurance** pour ce protocole de recherche : Responsabilité civile pour les recherches biomédicales (police n° 131.664) auprès de la société SHAM, Société Hospitalière d'Assurances Mutuelles, située 18 rue Edouard Rochet – 69372 LYON cedex 08, téléphone : 04.72.75.50.25.

Ce document d'information a pour but de vous expliquer exactement ce que cette étude implique afin de vous permettre de décider si vous voulez y participer.

Le Docteur .... discutera avec vous tout point qui ne vous semblerait pas clair et répondra à vos questions. N'hésitez pas à l'interroger afin d'obtenir plus de détails.

Informations Générales

Objectif général de cette étude

L'objectif de cette étude est d'évaluer l'efficacité d'une thérapie par un magnétiseur sur la disparition d'une verrue aux mains ou aux pieds, en 3 semaines après l'intervention.

Contexte

Vous avez une ou plusieurs verrues à la main ou au pied depuis au moins trois mois.

Les verrues sont une maladie fréquente de la peau, touchant le plus souvent les mains et les pieds, provoquée par le papilloma virus. Si les verrues ne sont pas dangereuses et disparaissent généralement avec le temps, sans traitement, elles peuvent être disgracieuses et douloureuses.

Il existe plusieurs traitements locaux des verrues de la peau, les plus utilisés sont une préparation contenant de l'acide salicylique et la cryothérapie (froid).
Beaucoup de patients consultent des magnétiseurs pour les verrues, il nous parait donc intéressant d'évaluer l'efficacité de cet acte. Actuellement, il n'existe aucune littérature sur le sujet permettant de conseiller les patients. C'est pourquoi nous souhaitons mener cette recherche clinique qui nous permettra peut-être de répondre à la question de l'efficacité de la prise en charge par un magnétiseur.

C'est la raison pour laquelle il vous est proposé de participer à une étude qui vise à évaluer l'efficacité du magnétisme sur le traitement des verrues.

Depuis toujours, les guérisseurs sont prêts à soigner, et deux français sur trois auraient déjà fait appel à eux au moins une fois au cours de leur existence. Beaucoup d'entre eux soignent en toute bonne foi en exerçant leur magnétisme, un « don » souvent hérité d'un aïeul. Ils suscitent à présent l'intérêt de médecins qui n'hésitent pas dans certains cas à s'assurer leur collaboration, à reconnaître implicitement leurs mystérieux talents.
Il nous semble utile d'explorer l'efficacité de cette technique selon une méthodologie fiable en étudiant une pathologie dont la guérison est objective.

Réalisation de l'essai

Cette étude est promue par le CHU de Caen.
Soixante-deux sujets au total seront inclus, pour comparer de 2 proportions observées :

- Le pourcentage de guérison dans le groupe traité par un "vrai" magnétiseur (reconnu de la profession)
- Le pourcentage de guérison dans le groupe traité par un "faux" magnétiseur (personne évaluée par un magnétiseur comme n'ayant pas de capacité à magnétiser). La personne «faux» magnétiseur, aura été formée à répliquer la gestuelle du vrai magnétiseur.

Les sujets seront recrutés via le Centre de Recherche Clinique (CRC) du CHU de Caen.
Lors de votre première visite au CRC (VS), vous serez reçu en consultation par un dermatologue et recevrez les informations de l'étude avant de signer le consentement de participation.
Le diagnostic de verrue sera confirmé par un examen clinique par le dermatologue.

Le type de traitement qui vous sera administré sera décidé par la méthode dite de randomisation (tirage au sort) par un processus automatisé par ordinateur. Vous aurez une probabilité sur deux d'être soigné par le "vrai" magnétiseur aussi bien que par le "faux" magnétiseur.
Vous serez questionné par le dermatologue sur votre croyance en cette méthode de magnétisme.
A l'issue de cette première visite, l'intervention par le magnétiseur sera programmée dans les jours qui suivent.

Lors de votre seconde visite au CRC, pour la visite dite (S0), vous serez pris en charge par le magnétiseur pour une séance d'environ 40 minutes. Cette séance consistera en une apposition des mains du magnétiseur sur le visage, les épaules, le dos, puis sur la ou les verrues. La verrue mère sera désignée et photographiée (c'est la photographie dite « à S0 »). Vous pourriez ressentir de la chaleur, du froid, ou encore des étourdissements. Le magnétiseur vous demandera de surveiller l'évolution de la verrue mère.
Après cette intervention, vous rentrerez à domicile. Vous ne devrez pas traiter votre verrue avec d'autre médicaments ou produits pendant cette période de trois semaines.

Trois semaines après l'intervention, vous serez revu au CRC : la verrue sera photographiée (photographie dite S3). Vous devrez nous rapporter les effets indésirables que vous auriez pu ressentir pendant ou après l'intervention du magnétiseur.

Six semaines après l'intervention, vous serez contacté par téléphone pour connaitre l'évolution de votre verrue. Il vous sera demandé de nous rapporter d'éventuels effets indésirables et traitements concomitants pris.

Les photographies des verrues à S0 et S3 seront évaluées à la fin de l'étude par le dermatologue qui analysera l'évolution de la verrue pendant cette période (guérison ou non). Ni vous ni le dermatologue, ne saurez si vous avez été pris en charge par le vrai ou le faux magnétiseur. Cette procédure dite en double aveugle est le seul moyen reconnu sur le plan scientifique pour obtenir des résultats fiables et donc démontrer l'efficacité de l'intervention.

Si les résultats étaient en faveur du magnétisme, cette étude pilote encouragerait des recherches supplémentaires pour savoir si la pratique du magnétisme peut constituer une spécialisation paramédicale.

Durée de participation

Il se déroulera six semaines entre le consentement et le dernier acte lié à la recherche.

Modalités de prise en charge médicale

Vous pourrez interrompre votre participation à la recherche si vous le désirez, à tout moment et quelle qu'en soit la raison.

Affiliation à un régime d'assurance maladie et examen médical préalable

Dans la cadre de votre participation à cette recherche biomédicale, nous vous informons que vous avez l'obligation d'être affilié à ou de bénéficier d'un régime d'assurance maladie.

Période d'exclusion et indemnité compensatrice

Il n'est pas prévu de période d'exclusion ni d'indemnité compensatoire pour cette étude.
Les séances de magnétisme sont gratuites dans le cadre de votre participation.

Respect du droit à l'image

Les photos de verrues pourront être utilisées pour des communications, mais votre anonymat sera respecté.

Risques prévisibles et effets secondaires associés à ce traitement

Aucun risque n'est à anticiper. A l'occasion de la consultation avec le dermatologue, un rendez-vous sera pris après la sortie d'étude dans un délai acceptable afin que vous receviez une alternative thérapeutique en cas de non efficacité, traitement par cryothérapie (par le froid) ou par une préparation à base d'acide salicylique par exemple. Ce délai nous semble acceptable compte tenu de la faible gravité des verrues.

Bénéfices attendus

Le bénéfice individuel attendu de la recherche n'est pas évaluable puisqu'il s'agit d'un sujet de recherche qui n'a pas été évalué à notre connaissance dans la littérature dans cette pathologie. Néanmoins, notre revue de la littérature nous a permis de recenser les études sur les thérapies alternatives dans d'autres indications que la verrue : certaines ont montré une efficacité et aucune n'a rapporté de problème de tolérance.
Le bénéfice collectif sera selon le résultat : en cas d'effet nul ou défavorable du magnétisme, cette alternative ne devra pas être retenue par les médecins. En cas de réponse positive, des recherches

complémentaires pourront être menées pour valider l'intérêt du magnétisme dans cette pathologie voire dans d'autres.

Participation

Votre participation à ce projet de recherche est volontaire. Vous avez le droit de refuser de participer à cette recherche sans avoir à vous justifier, ou d'arrêter le traitement à tout moment en cours d'étude sans aucun préjudice (sans conséquences sur la suite de votre prise en charge) pour vous. Votre décision n 'affectera en aucune façon la prise en charge ultérieure prodiguée par votre médecin.

Si vous ne vous présentez pas aux examens médicaux prévus ou si vous ne respectez pas les exigences du protocole ou les instructions de votre médecin, ce dernier pourra décider d'interrompre votre participation à l'étude.

Vous avez bien entendu la possibilité de réfléchir, avant de prendre votre décision ou de refuser sans que cela ne modifie en quoi que ce soit vos relations avec l'équipe médicale. Ce document vous appartient et nous vous invitons à en discuter avec votre médecin traitant et vos proches. Vous avez le droit de retirer votre consentement à tout moment sans avoir à vous justifier.

L'investigateur principal vous tiendra informé de toute information nouvelle survenant pendant votre participation et pouvant influencer sur votre décision de participation.

Dans cette étude les données nominatives seront enregistrées, transmises et centralisées à l'Unité de Biostatistique et Recherche Clinique du Centre Hospitalo-Universitaire de Caen. Ces données démographiques, cliniques, biologiques, thérapeutiques seront analysées par des moyens informatiques, respectant le secret statistique et médical.

L'analyse statistique de vos données permettra d'évaluer l'efficacité d'une thérapie par un magnétiseur sur la disparition d'une verrue vulgaire palmaire ou plantaire à 3 semaines d'une prise en charge par le magnétiseur.

La mise en évidence de relation(s) éventuelle(s) repose sur les méthodes statistiques appropriées. Ces analyses effectuées par un médecin statisticien du CHU de Caen.

Aspects légaux

Dans le cadre de la recherche biomédicale à laquelle le CHU de Caen vous propose de participer, un traitement de vos données personnelles va être mis en œuvre pour permettre d'analyser les résultats de la recherche au regard de l'objectif de cette dernière qui vous a été présenté.

A cette fin, les données médicales vous concernant seront transmises au promoteur de la recherche ou aux personnes ou sociétés agissant pour son compte, en France ou à l'étranger. Ces données seront identifiées par un numéro de code et/ou vos initiales ou les trois premières lettres de votre nom. Ces données pourront également, dans des conditions assurant leur confidentialité, être transmises aux autorités sanitaires de santé ou judiciaires.

Conformément aux dispositions de la loi relative à l'informatique aux fichiers et aux libertés (en conformité avec la loi n° 2004-801 du 6 août 2004 relative à la protection des personnes physiques à l'égard des traitements de données à caractère personnel et modifiant la loi n° 78-17 du 6 janvier 1978 relative à l'informatique, aux fichiers et aux libertés), vous disposez d'un droit d'accès et de rectification des fichiers contenant vos données personnelles. Vous disposez également d'un droit d'opposition à la transmission des données couvertes par le secret professionnel susceptibles d'être utilisées dans le cadre de cette recherche et d'être traitées.

Vous pouvez également accéder directement ou par l'intermédiaire d'un médecin de votre choix à l'ensemble de vos données médicales en application des dispositions de l'article L1111-7 du Code de la Santé Publique. Ces droits s'exercent auprès du médecin qui vous suit dans le cadre de la recherche et qui connaît votre identité.

Conformément à la loi n° 2002-303 du 4 mars 2002 relative aux droits des malades et à la qualité du système de santé, le CHU de Caen vous tiendra informé des résultats globaux de cette Recherche à la fin de l'étude.

Si vous acceptez de participer à cette étude, nous vous demandons de bien lire attentivement le formulaire de consentement joint et de le signer. Ce consentement ne dégage en rien la responsabilité de l'investigateur et du promoteur. Vous avez la possibilité de poser des questions à tout moment avant et en cours d'étude auprès de :

Dr / Pr ....

Tel ....

(en cas d'indisponibilité, vous pouvez contacter l'investigateur principal, le Dr Dompmartin, au 02.31.27.25.10, Mail : dompmartin-a@chu-caen.fr).

Vous pouvez interrompre à tout moment votre participation à l'étude sans que cela ne porte atteinte aux bons soins qui vous sont prodigués, ni à vos relations avec votre médecin.

Cette recherche a reçu l'avis favorable du Comité de Protection des Personnes (CPP) Nord-Ouest III, situé au CHU de Caen, niveau 03 – porte 03-508, avenue de la côte de nacre – 14033 CAEN cedex 05 le 05/12/2015 et l'autorisation de l'autorité compétente (Agence Nationale de Sécurité du Médicament et des produits de santé : ANSM) le 04/11/2015.

Consentement éclairé

Je, soussigné(e), ………………………………………déclare participer volontairement à la recherche biomédicale intitulée :

Titre de la recherche : « protocole MAGNETIK - Effet de l'intervention d'un magnétiseur sur les verrues des mains et des pieds des adultes : essai prospectif randomisé versus placebo en double aveugle. »

Ce après avoir été informé des implications engendrées par ma participation par l'investigateur principal de cette recherche.

Le promoteur est le CHU de Caen, avenue de la côte de nacre – 14033 CAEN, téléphone : 02.31.06.57.81.

L'investigateur coordinateur de l'étude est le Dr Dompmartin, service de dermatologie au CHU de Caen, avenue de la côte de nacre – 14033 CAEN, téléphone : 02.31.27.25.10.

L'étude clinique est référencée sous le numéro **ID RCB n° 2015-A01609-40**.

Le promoteur de l'étude a souscrit une **assurance** pour ce protocole de recherche : Responsabilité civile pour les recherches biomédicales (police n° 131.664) auprès de la société SHAM, Société Hospitalière d'Assurances Mutuelles, située 18 rue Edouard Rochet – 69372 LYON cedex 08, téléphone : 04.72.75.50.25.

J'ai reçu et compris les informations reportées dans le document d'information, ci-joint, qui m'a été également remis. J'ai été informé(e) du but, de la nature, de la durée, des bénéfices et des risques prévisibles de l'étude et ce que l'on attend de moi. J'ai pu poser toutes les questions que je voulais et j'ai reçu des réponses adaptées

J'ai disposé d'un temps de réflexion suffisant entre l'information et le consentement.

J'ai bien compris que j'ai le droit de refuser de participer ou de retirer mon consentement à tout moment sans avoir à me justifier et que ma décision sera sans conséquence sur mes relations futures avec l'investigateur et les soins qui me seront dispensés.

J'accepte de façon libre et volontaire de participer à la présente recherche.

J'ai compris que les données médicales me concernant resteront confidentielles et ne pourront être consultées que par l'investigateur et ses collaborateurs ou par des personnes mandatées par le promoteur et astreintes au secret professionnel ou par des personnes mandatées par les autorités sanitaires et judiciaires.

J'accepte le traitement informatisé des données nominatives en conformité avec la loi n° 2004-801 du 6 août 2004 relative à la protection des personnes physiques à l'égard des traitements de données à caractère personnel et modifiant la loi n° 78-17 du 6 janvier 1978 relative à l'informatique, aux fichiers et aux libertés.

Le présent consentement ne dégage pas le promoteur et les investigateurs de leurs responsabilités.

Je pourrai à tout moment avoir accès aux données de l'étude et le promoteur me tiendra informé en fin d'étude des résultats globaux de cette Recherche, selon la loi n° 2002-303 du 4 mars 2002.
Je pourrai exercer mon droit d'accès et de rectification auprès de l'investigateur coordinateur de la recherche, le Dr Dompmartin, service de dermatologie au CHU de Caen, avenue de la côte de nacre – 14033 CAEN, téléphone : 02.31.27.25.10.

Signature du médecin qui atteste avoir pleinement expliqué à la personne signataire le but, les modalités ainsi que les risques potentiels de la recherche

Nom, prénom :____

Date |__|__||__|__||__|__|__|__| **Signature**

Signature de la personne donnant son consentement

Nom, prénom: ____
____

Date |__|__||__|__||__|__|__|__| **Signature**

Lieu
